# Patient Bridge Role: a new approach for patient and public involvement in healthcare research programmes

**DOI:** 10.1136/bmjopen-2024-094521

**Published:** 2025-05-15

**Authors:** Beccy Summers, Leon Farmer, Sharon Cooper, Christian von Wagner, Bettina Friedrich, Gary A Abel, Anne Spencer, Emma Cockcroft

**Affiliations:** 1 Applied Research Collaboration South West Peninsula, Patient and Public Involvement and Engagement Team, University of Exeter, Exeter, UK; 2 PPIE, University of Exeter Medical School, Exeter, UK; 3 University of Exeter Faculty of Health and Life Sciences, Exeter, UK; 4 Department of Epidemiology and Public Health, UCL, London, UK; 5 University College London, London, UK; 6 University of Exeter Medical School (Primary Care), University of Exeter, Exeter, UK; 7 Health Economics, University of Exeter Medical School, Exeter, UK; 8 Clinical Trials Unit, University of Exeter Medical School, Exeter, UK

**Keywords:** Patient Participation, Patients, Primary Health Care

## Abstract

**Abstract:**

**Background:**

Patient and public involvement (PPI) in research involves an active collaboration between patients/members of the public and researchers in equal partnership. PPI in health research ensures the research benefits those most impacted by the research and is a well-established necessity of high-quality research. PPI for large programmes of work involving multiple studies frequently relies on a single PPI group that oversees the entire programme. We believe that this ‘traditional’ approach can negatively contribute to the power imbalance between researchers and PPI members, since PPI members have a very wide remit and are unable to embed themselves fully in all aspects of the research.

**Aim:**

The study aimed to evaluate a novel PPI approach, the ‘Patient Bridge Role’, designed to promote a more equal distribution of power between public collaborators and researchers in a large research programme. The Patient Bridge Role involves assigning specific public collaborators to each work package, facilitating deeper engagement and communication.

**Main argument:**

The Patient Bridge Role addresses the limitations of traditional PPI. This approach requires clear role definitions and collaborative development of guidelines to ensure effective communication and shared decision-making. Despite initial challenges related to role clarity and boundaries, the Patient Bridge Roles successfully promoted a more balanced partnership between researchers and public collaborators.

**Conclusions:**

Active partnerships between public collaborators and researchers are critical to creating more relevant and higher quality research. Yet, there are many practical and conceptual barriers to this. The Patient Bridge Role offers a promising strategy for enhancing PPI in large research programmes.

## Background

 Patient and public involvement (PPI) in research involves an active partnership and collaboration between public collaborators (ie, patients and members of the public) with researchers. PPI ensures that research benefits those it intends to, is relevant and conducted in an acceptable and appropriate way. PPI in health research is a well-established necessity of quality research.^1^ In 2022, funders, regulators as well as other organisations involved in delivering research in the UK signed the Shared Commitment to Public Involvement, which is a commitment to embed PPI in research.^2^

There is a lack of consensus on what constitutes ‘effective’ involvement.^3^ Several models and frameworks exist to help guide researchers in best practice for involving patients.^4^ Despite recommendations and guidelines, the degree to which patients and the public are involved in health research varies greatly. A realist evaluation of 478 non-commercial studies conducted between 2009 and 2011 within the UK identified that 49% of studies had no evidence of PPI.^5^ A more recent cross-sectional analysis determined that out of 3000 health research papers published internationally in 2020, 79.4% of research papers did not include reporting of PPI.^6^ Even when PPI is included within studies, patients and the public are often merely consulted about the research rather than active and equal partners (table 1—levels of PPI).^7^

**Table 1:** The ladder of coproduction (adapted from National Co-production Advisory Group^24^)

Coproduction	Equal relationship between patients, public and researchers exists, and you work together from designing to delivering, sharing strategic decision-making about the research.
Codesign	Patients and public are involved in designing research based on their experiences and ideas. They have a genuine influence but are not involved in ‘seeing it through’.
Engagement	Where patients and public are given opportunities to express their views about the research and are able to influence some decisions that are made about that research.
Consultation	Patients and public are asked to share their perspective on the researcher. However, this step may be considered tokenistic if patients and public don’t have the power to influence change.
Informing	Inform patients and public about the research including telling them what decisions have been made and why.
Educating	Patients and public are helped to understand the design and delivery of the research so that they gain relevant knowledge about it.
Coercion	Patients and public to be passive recipients of information about research but their views are not considered important and not being taken into account.

### Barriers to collaboratively working with public collaborators

Several practical and conceptual barriers to collaborative working with public collaborators have previously been reported.^8 9^ One of the most prominent practical barriers for researchers engaging in collaborative PPI is the availability of funds.^10^ The development of a research idea into a fully fledged grant application is a prime point at which to involve patients and the public so that the project is relevant to a diverse range of service-users.^11^ However, this stage of the research cycle is often undertaken without any funding. This impacts negatively on the diversity of public collaborators. For example, not having funds for translators, to cover travel or childcare costs, means that only some people will be able to take time out to input on the research.^9^

One of the most prominent barriers, and the focus of this paper, is the power relationship between public collaborators and researchers.^9^ One of the main principles of co-production is the need for researchers and public collaborators to share power and influence on decision-making. If the research is jointly owned, and people work together to achieve a joint understanding, experiential knowledge and expert knowledge can come together to equally inform the research.^12^ However, a lack of willingness and/or ability persists among researchers to adopt this shared power stance with public collaborators.^6 8 13 14^ Instead, researchers fall back on more ‘traditional’ views of PPI where they hold the decisional power and public collaborators remain passive recipients of information about the project.^6 8 14^

Embedding involvement within large programmes of work can present further challenges. Large programmes of work generally comprise a number of high-quality interrelated projects, usually described in separate work packages (WPs), that form a coherent theme, where added value is gained from the combination of the various strands of research.^15^ There are only a limited number of studies reporting on the process and examples of PPI in large programme grants.^16 17^ However, they often rely on having a single PPI group that oversees the entire programme of work, similar to that of smaller studies. While it would never be expected for all researchers from one WP to be fully immersed in the work of all the other packages within the programme, the traditional approach of having a single PPI group does expect public collaborators to work in this way. As such, this approach to PPI can jeopardise the chance for public collaborators to be equally involved in shared decision-making in large programmes of work.

This paper examines and critically reflects on the realities, challenges and learning from the implementation of a new approach to PPI that enables a more equal distribution of power between public collaborators and researchers, facilitating wider shared decision-making in similar multiphase projects.

#### Involvement context

The Spotting Cancer among Comorbidities Project (SPOCC) is a 5-year National Institute for Health Research (NIHR) funded research programme (Award ID: NIHR201070). It was developed to investigate if, when and how the diagnosis of cancer is affected for people with one or more pre-existing health conditions.^18^ The research programme is exploring which patients with pre-existing health conditions are likely to have a cancer diagnosis happen more slowly and why. The ultimate aim of the programme is to develop a new tool to help achieve a timelier diagnosis of cancer in patients with pre-existing health conditions.

To reach this aim, six WPs are being carried out over the 5 years (table 2—WP within the SPOCC programme). The programme is currently in its third year.^18^

**Table 2:** Work packages (WP) within the SPOCC programme

WP 1	Using existing data from general practice and The National Cancer Registration and Analysis Service, we will identify which health conditions are associated with delayed cancer diagnosis.
WP 2	We will interview both patients with pre-existing conditions and GPs to understand their experience and views of spotting potential cancer symptoms in the presence of pre-existing medical conditions.
WP 3	Using a survey which will include imaginary situations, we will try and understand how patients, GPs and nurses respond to and make decisions about potential cancer symptoms in the presence of other conditions.
WP 4	We will use existing data on symptom reporting and cancer diagnosis to estimate the risk of potential cancer for patients with different conditions and symptoms to develop risk assessment tools. These tools could improve how cancer is diagnosed in patients with pre-existing medical conditions.
WP 5	With the help of the knowledge gained in the studies 1–4, and collaborating with patient and clinicians, we will develop and test a new approach aimed to reduce the delay in diagnosing cancer in patients with pre-existing conditions.
WP 6	We will look at the cost of this new approach and its ‘value for money’ in terms of improving cancer diagnosis and care of patients.

GP, general practitioner; SPOCC, Spotting Cancer among Comorbidities.

## Planned involvement methods

**Figure 1:**
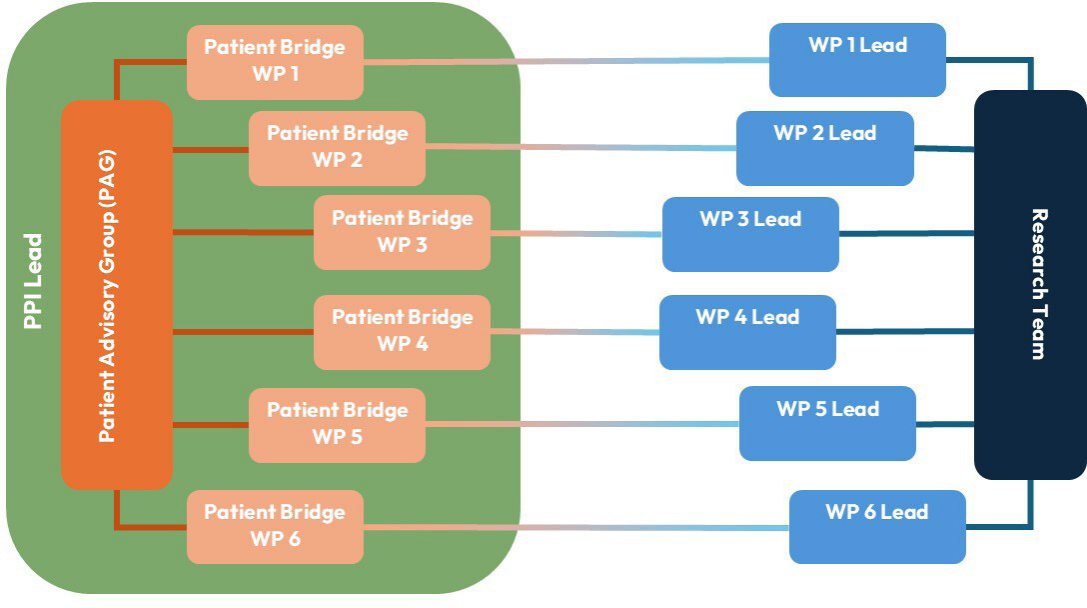
Proposed involvement context of the SPOCC programme. The PAG will consist of approximately eight people who have experience of living with cancer and/or other selected long-term health conditions. Each of the WPs within the programme will have a named PAG contacted, termed the Patient Bridge. The Patient Bridge will ensure that there is clear communication between their WP and the PAG. Each Patient Bridge will be supported by the PPI lead on the programme. PAG, Patient Advisory Group; PPI, patient and public involvement; SPOCC, Spotting Cancer among Comorbidities; WP, work packages.

Our proposed approach for PPI in research was planned to prevent a power imbalance developing between researchers and public collaborators, so that wider shared decision-making could take place in the programme. Our proposed approach consisted of a Patient Advisory Group (PAG), composed of approximately eight people who have experience of cancer and/or other selected long-term conditions such as type 2 diabetes or chronic obstructive pulmonary disease. The group was proposed to meet approximately three times per year to discuss specific aspects of each WP including, but not limited to, finalising protocols, coproducing patient-facing documents, informing analysis and interpreting data, intervention development and dissemination. The innovative feature of our new approach to working (figure 1) was that each WP would have a named contact, termed the Patient Bridge, from within the PAG. Not every public collaborator from within the PAG took on a Patient Bridge Role, but all Patient Bridges were members of the PAG. Both the PAG and Patient Bridges were supported by the PPI lead on the programme. The Patient Bridge could liaise directly with WP leads regarding PPI in the WP. This enabled the Patient Bridge for each WP to develop a deeper understanding of their specific WP. The deeper knowledge of the WP contributed significantly to Patient Bridge’s feeling of an equal distribution of power between themselves and the WP leads.

The initial outline of the Patient Bridge Role was intentionally broad, to evolve the role as public collaborators and the researcher team saw fit as the programme progressed. However, our new approach to working did present two main challenges relating to the specifics and the boundaries of the Patient Bridge Role. The next section will summarise the challenges faced and the actions taken to overcome them.

## Challenge one: specifics of the Patient Bridge Role

During initial meetings, it was evident that researchers were unclear how the Patient Bridge could facilitate updates from the researchers to the PAG. The PPI lead was asked by researchers on numerous occasions “when should I contact the Patient Bridge?”. As a result, the PAG felt left “out of the loop” and not valued, which began to erode the mutual respect that was once felt between the public collaborators and the researchers. This began to create a power imbalance between the researchers and the public collaborators, leading to a climate in which shared decision-making on the research would not flourish.^12^

To overcome this challenge, the PAG discussed with the PPI lead proactive ways to re-establish a more equal power balance. The PPI lead suggested that providing the research team with worked examples would be a robust approach to take.^19^ Therefore, the PAG and PPI lead coproduced worked examples of how researchers and the Patient Bridge could work together to facilitate updates from the researcher to the PAG. The final worked example (ie, a comic strip) (online supplemental file 1) relied on images to convey the message as these attract people’s attention, as well as aid learning and recall.^20^ The researchers were not simply passive recipients of the worked example. Instead, once they had taken onboard the learning regarding the Patient Bridges’ expectations, a discussion session was held for researchers to contribute their perspective and expectations. This approach ensured that the Patient Bridges’, the PAG and the researchers’ knowledge were all valued and respected, thereby upholding the principles of coproduction.^12^

## Challenge two: boundaries of the Patient Bridge Role

The second challenge that arose in the programme was difficulty understanding what fell within the Patient Bridge Role and what fell within the role of the PPI lead. This in turn meant that researchers were somewhat hesitant to engage with the Patient Bridge because they were unsure whether their requests would be appropriate. The issue of boundaries is perhaps not a unique challenge to this programme because there is a wealth of evidence demonstrating researchers’ uncertainty regarding the appropriate ways to engage with public collaborators.^8 21^ At the start of the programme, the role of the Patient Bridge was not rigidly defined because it was critical that public collaborators and researchers were able to evolve this new role as they saw fit within the programme. Although this open approach was indeed in the spirit of coproduction, it meant that as the role was evolving, researchers had no clear parameters in terms of the scope of the role, that could be used as a point of reference when engaging with the Patient Bridge.

When people working in a team are not clear what their responsibilities and role are, they are not productive. There is confusion, disappointment and often frustration.^22^ It was evident that these negative impacts were beginning to emerge within this programme, acting as barriers to meaningful PPI. However, as the programme had progressed, the public collaborators began to have a clearer vision of how they wanted to be involved in the programme. This generated the idea within the PAG that a coproduced role description for the Patient Bridge Role could overcome the challenge of a lack of clearly defined roles. As such, the PAG and the PPI lead set about coproducing the role description, which had at its core ensuring that everyone involved in the project had clearly defined roles and held real responsibilities to ensure that there was shared power and ownership of the project among public collaborators and researchers.^12^

Purposefully, the role description was designed to empower public collaborators in their Patient Bridge Role.^12^ As a part of this, more power was placed in the hands of the Patient Bridge Role as a PPI facilitator instead of solely in the hands of the PPI lead.^12^ The Patient Bridge Role description contained a clearly defined purpose for the role: “ensures that there is clear two-way communication between WP researchers and the PAG enabling robust PPI input throughout the SPOCC programme”. It also contained the key responsibilities and duties of the Patient Bridge (table 3) as well as the key support responsibilities and duties of researchers and the PPI lead (table 4).

**Table 3:** Key responsibilities and duties of the Patient Bridge within the SPOCC programme

Key responsibilities and duties of the Patient Bridge	Work in collaboration with WP team	Establish and adhere to an agreed updating procedure with WP lead that enables progress reports to be regularly provided to Patient Bridge (eg, attend WP team meetings or monthly email exchange or monthly Patient Bridge WP lead meeting).
		Clearly and promptly communicate researchers’ questions to the PAG.
		Share own lived experience in a productive way that helps inform the WP and its outputs.
	Work in collaboration with PAG and PPI lead:	Provide brief progress report on WP to PPI lead ahead of PAG meetings.
		Constructively report any concerns regarding clear and prompt communication between Patient Bridge and WP lead to PPI lead.
		Clearly and promptly communicate items for the PAG meeting agenda ahead of PAG meetings to the PPI lead.
		Regularly attend and engage with PAG meetings.
		Provide clear and concise progress reports/updates on WP at PAG meetings.
		Clearly and promptly communicate PAG input to WP researchers.
		Clearly and promptly communicate PAG questions to WP researchers.

PAG, Patient Advisory Group; PPI, patient and public involvement; SPOCC, Spotting Cancer among Comorbidities; WP, work packages.

**Table 4:** Key responsibilities of researchers and PPI lead within the SPOCC programme

Key support responsibilities of researchers	1. Establish and adhere to an agreed updating procedure with WP Patient Bridge that enables progress reports to be regularly provided to Patient Bridge (eg, attend WP team meetings or monthly email exchange or monthly Patient Bridge WP lead meeting).
	2. Clearly and promptly communicate with Patient Bridge to draw on their lived experiences to inform the WP research and its outputs.
	3. Clearly and promptly raise questions to the Patient Bridge that you wish for them to take to the PPI group so that their lived experience can inform the WP research and its outputs.
	4. Provide timely and adequate space for Patient Bridge to feedback input from the PPI group and clearly communicate the impact that the PPI input has had on the WP to the Patient Bridge.
Key support responsibilities of PPI lead	1. Facilitate communication and collaboration between Patient Bridge and WP lead, ensuring to assist the Patient Bridge in tackling breakdowns/lapses in this process.
	2. Provide adequate training and advice where necessary so that Patient Bridge feels equipped to fulfil the responsibilities of their role.

PPI, patient and public involvement; SPOCC, Spotting Cancer among Comorbidities; WP, work packages.

**Figure 2:**
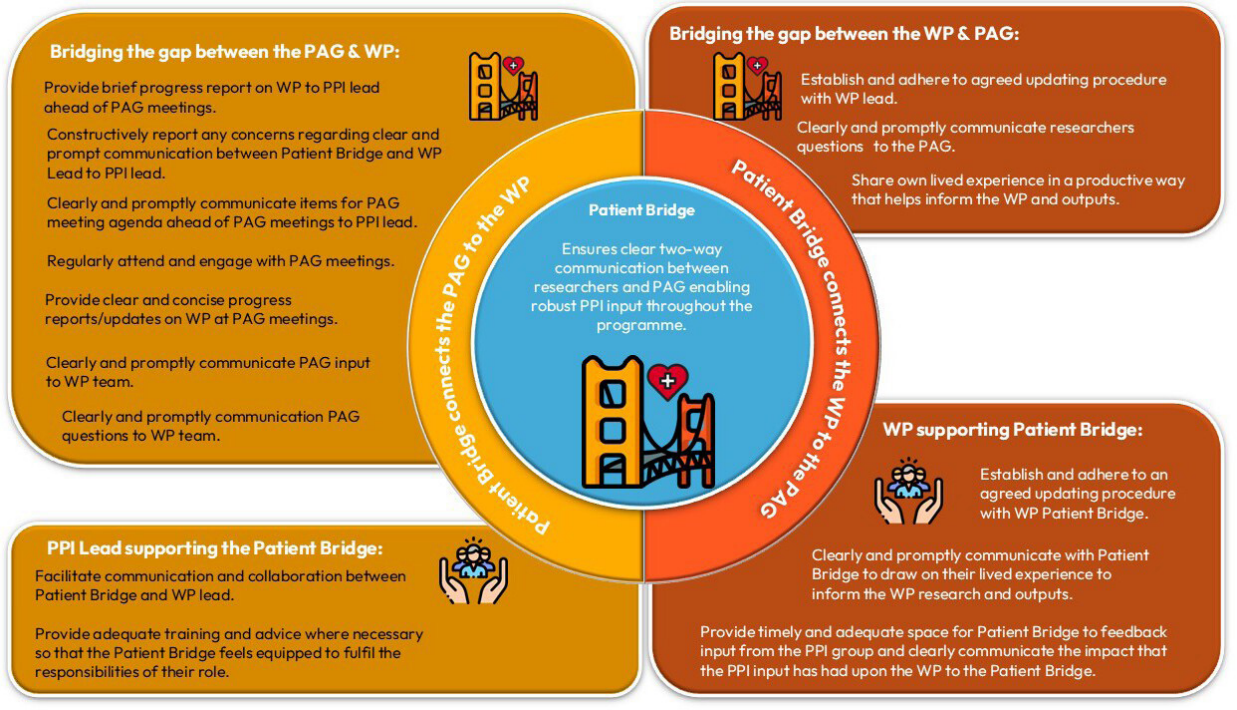
Patient Bridge Approach. The Patient Bridge (defined within centre blue circle) has two core roles. The first core role is connecting the PAG to the WP, which the Patient Bridge achieves by performing the actions listed (top left yellow rectangle). The PPI lead supports the Patient Bridge in bridging the gap between the PAG and WP by conducting the actions listed (bottom left yellow rectangle). The second core role of the Patient Bridge is connecting the WP to the PAG, which the Patient Bridge achieves by conducting the actions listed (top right orange rectangle). The WP researchers support the Patient Bridge in bridging the gap between the WP and the PAG by conducting the actions listed (bottom right orange rectangle). PAG, Patient Advisory Group; PPI, patient and public involvement; WP, work packages.

The role description was shared with the research team to ensure that the document was coproduced with their input as well. After a time of discussion, the role description was approved for use in the programme. The implementation of the role description had an immediate positive impact on the programme. It supported the specialisation of the Patient Bridge to their WP, akin to the specialisation of the research lead, enabling the Patient Bridge to embed themselves fully in all aspects of the WP. Public collaborators reported that it generated increased collaboration between themselves and researchers. Additionally, researchers reported that they felt more prepared to approach interactions with public collaborators as they were clear about public collaborators’ expectations of them. As a result, researchers felt equipped to seize more opportunities for PPI within the programme. For example, the Patient Bridges from WP5 and WP6 collaborated with each other and the WP6 lead to help dovetail the plans and implementation of these two related WPs. Figure 2 contains the final Patient Bridge role that has been developed that enabled a more equal distribution of power between public collaborators and researchers, which facilitated wider shared decision-making within the programme.

### Learning to take forward in future research

There is sparse literature focused on exploring approaches to PPI aimed at promoting a more equal distribution of power between public collaborators and researchers within large programmes of work. The approach that this small evidence base favours is the integration of multiple PPI groups within a programme.^17 23^ This indeed has benefits; for example, it enables groups to be established around different language needs, thereby supporting a more inclusive approach.^23^ However, multiple PPI groups within a programme still means there is an expectation that each group will be fully immersed in each WP within the programme. As such, this approach can still jeopardise the chance for public collaborators to be equally involved in shared decision-making. Whereas the Patient Bridge role detailed in this paper offers a novel way to support a more equal distribution of power between public collaborators and researchers, facilitating wider shared decision-making in large programmes.

Experiences gained from working through the challenges highlight four important points of learning to take forward. First, we encourage research teams and public collaborators to take on new approaches to PPI and work together to adjust these because these processes of adjustment have the potential to lead to a more equal working relationship between public collaborators and researchers. This creates the conditions for more shared decision-making throughout the research. Second, the flexibility within the research funding that allowed for flexibility regarding the PPI approach enabled the development of this approach and for people to try, fail and make it better. Additional scope for embedding PPI evaluation within programmes will enable new and potentially more promising approaches to PPI to be coproduced. Third, ensuring the roles and ways of working are shared internally in an accessible way so that they are more easily grasped and engaged with by the team is critical. Fourth, this Patient Bridge role only works when the whole team, researchers, public collaborators and PPI facilitators, are committed to this approach of establishing an equal power relationship between public collaborators and researchers.

## Conclusions

Active partnerships between public collaborators and researchers are critical to creating more relevant and higher quality research. Yet, there are many practical and conceptual barriers that stand in the way. This paper has detailed our innovative Patient Bridge role which, unlike more traditional approaches to PPI, enables public collaborators and researchers to work as equal partners within large programmes of work. The development of this approach was not without its challenges; however, these provided important learning points to take forward in future practice.

## Supplementary material

10.1136/bmjopen-2024-094521 online supplemental file 1
