# Supplementary material for: Patient Bridge Role: a new approach for patient and public involvement in healthcare research programmes
Source: BMJ Open. 2025 May 15;15(5):e094521. doi: 10.1136/bmjopen-2024-094521 (PMC12083370; doi:10.1136/bmjopen-2024-094521)

Patient Bridge Role: In Action
Agreeing a collaborative approach

I need input from the WP Patient Bridge. I've not worked with them before so we should agree how best to work together first.

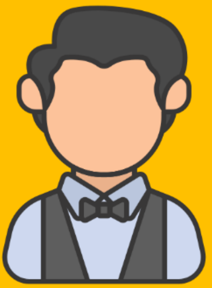

Hi Elizabeth,

I'm looking forward to working with you on this WP. I thought first it would be best to agree upon how we will work together.

How do you prefer I contact you for WP updates and for points of discussion/questions that I have?

How often would you like updates on the WP?

Are there any additional needs I need to meet for you?

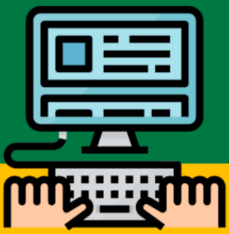

Hi Barry,

Great to hear from you!

It would be great if you could ,continue with email to contact me as I'm more comfortable taking my time to write down my thoughts/comments.

I would like to have updates on the WP once a month. I'm happy to have a brief email update or attend a WP team meeting to get the update.

It is easier for me to read documents that have a dark background with light writing, so it would be great if you could keep that in mind when you send me things to read.

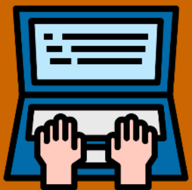

Hi Elizabeth,

Brilliant – I'll make sure to reach out to you by email and to ensure I send appropriately coloured documents to you.

No problem, I'll send brief updates via email once a month and it would be good to have you at team meetings when there are discussion points we would like your input on. Would you like to attend our next team meeting to meet the team and get your first update on the WP?

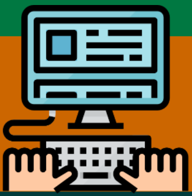

Hi Barry,

Great – thanks for attaching the agenda.

See you there!

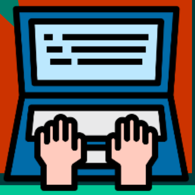

Morning everyone, we have Elizabeth with us today who is the Patient Bridge for this WP. Let's all go around and introduce ourselves before we give Elizabeth an update of WP progress.

Hiya Elizabeth, I'm Joe great to be working with you!

Hiya Elizabeth, I'm Iris, great to meet you! I can give you an update……

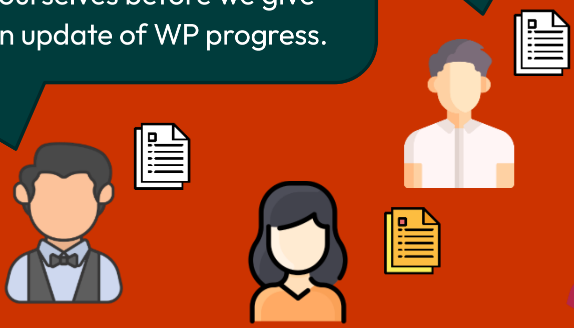

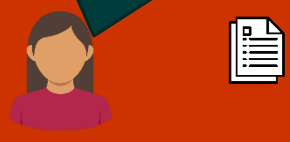

Supplement: online supplemental file 1 [file bmjopen-15-5-s001.pdf]
