# Supplementary material for: Patient Bridge Role: a new approach for patient and public involvement in healthcare research programmes
Source: BMJ Open. 2025 May 15;15(5):e094521. doi: 10.1136/bmjopen-2024-094521 (PMC12083370; doi:10.1136/bmjopen-2024-094521)
Supplement: Supplementary file 2 [file bmjopen-2024-094521.reviewer_comments.pdf]

PEER REVIEW HISTORY

BMJ Open publishes all reviews undertaken for accepted manuscripts. Reviewers are asked to complete a checklist review form and are provided with free text boxes to elaborate on their assessment. These free text comments are reproduced below.

ARTICLE DETAILS

Title (Provisional)

The Patient Bridge Role: A New Approach for Patient and Public Involvement in Healthcare Research Programmes.

Authors

Summers, Beccy; Cooper, Sharon; Farmer, Leon; von Wagner, Christian; Friedrich, Bettina; Abel, Gary A; Spencer, Anne; Cockcroft, Emma

---

VERSION 1 - REVIEW

**Reviewer**	**1**
**Name**	**Luke, Alison**
**Affiliation**	**University of New Brunswick, Faculty of Nursing and Health Sciences**
**Date**	**10-Feb-2025**
**COI**	**None**

Excellent article. Addresses a very important issue in patient oriented research and poses a creative solution to

---

**Reviewer**	**2**
**Name**	**Santana, Maria-Jose**
**Affiliation**	**University of Calgary, Community Health Sciences**
**Date**	**26-Mar-2025**
**COI**	**None**

Thanks to the authors for this addition to the PPI literature. This communication article brings up the importance of power imbalances when working together with patient and public in health research and presents a solution, the Patient Bridge.

This communication article discusses the importance on meaningful engagement of patents and public in health research. Specifically, the authors present the Patient Bridge as a potential solution to power imbalances when involving patients in health research projects.

When reviewing this manuscript this article raises the important issue of meaningful connection and communication to support PPI, and it's relevant to researchers and patients interested in working together in research. The concept of Patient Bridge is novel. There is evidence from previous work on the importance of avoiding power imbalances and tokenism approaches to PPI. The authors in this communication article presented evidence primarily from the UK and that's fine as it is a UK project. However, it would have been interesting to compare and contrast with other international practices. For instance, what are the patients and public in Canada proposed to overcome similar challenges (see a communication article by Santana, M.J., Duquette, D., Fairie, P. et al. Patient-identified priorities for successful partnerships in patient-oriented research. Res Involv Engagem **8**, 49 (2022). https://doi.org/10.1186/s40900-022-00384-4).

The article is a PPI well written with a clearly presented content. It will help to other researchers and patients interested in PPI to develop strategies to overcome barriers like power imbalances. It would have been interesting to learn about whether the Patient Bridge overcome completed power imbalances, and the details associated to it. If the authors could evaluated the Patient Bridge role, it will be terrific, and I look forward to reading the next piece.

VERSION 1 - AUTHOR RESPONSE

	Comment	Response
Reviewer 1	Excellent article. Addresses a very important issue in patient oriented research and poses a creative solution to.	Thank you for the kind comment.
Reviewer 2	Thanks to the authors for this addition to the PPI literature. This communication article brings up the importance of power imbalances when working together with patient and public in health research and presents a solution, the Patient Bridge	Thank you for the supportive comments.
